# Screening New Xylanase Biocatalysts from the Mangrove Soil Diversity

**DOI:** 10.3390/microorganisms9071484

**Published:** 2021-07-12

**Authors:** Corinne Ivaldi, Mariane Daou, Laurent Vallon, Alexandra Bisotto, Mireille Haon, Sona Garajova, Emmanuel Bertrand, Craig B. Faulds, Giuliano Sciara, Adrien Jacotot, Cyril Marchand, Mylène Hugoni, Harivony Rakotoarivonina, Marie-Noëlle Rosso, Caroline Rémond, Patricia Luis, Eric Record

**Affiliations:** 1 INRAE, FARE, UMR A 614, Chaire AFERE, Université de Reims Champagne Ardenne, 51097 Reims, France; corinne.ivaldi@univ-reims.fr (C.I.); Harivony.rakotoarivonina@univ-reims.fr (H.R.); caroline.remond@univ-reims.fr (C.R.); 2 INRAE, UMR1163, Biodiversité et Biotechnologie Fongiques, Aix-Marseille Université, 13288 Marseille, France; mariane.daou@ku.ac.ae (M.D.); alexandra.bisotto@inrae.fr (A.B.); mireille.haon@inrae.fr (M.H.); sona.garajova@inrae.fr (S.G.); emmanuel.bertrand@univ-amu.fr (E.B.); craig.faulds@univ-amu.fr (C.B.F.); giuliano.sciara@inrae.fr (G.S.); marie-noelle.rosso@inrae.fr (M.-N.R.); 3 Department of Chemistry, Khalifa University, Abu Dhabi 127788, United Arab Emirates; 4 CNRS, INRAE, VetAgro Sup, UMR Ecologie Microbienne, Université Lyon, Université Claude Bernard Lyon 1, F-69622 Villeurbanne, France; laurent.vallon@univ-lyon1.fr (L.V.); mylene.hugoni@univ-lyon1.fr (M.H.); patricia.luis@univ-lyon1.fr (P.L.); 5 Institut de Recherche pour le Développement (IRD), IMPMC, UPMC, CNRS, MNHN, 98851 Noumea, New Caledonia, France; adrien.jacotot@unc.nc (A.J.); cyril.marchand@unc.nc (C.M.); 6 ISEA, Université de la Nouvelle-Calédonie, EA 7484, 8 BPR4, 98851 Noumea, New Caledonia, France; 7 CNRS, BRGM, ISTO, UMR 7327, Université d’Orléans, 45071 Orléans, France

**Keywords:** lignocellulose degrading enzymes, xylanases, heterologous expression, biomass degradation, marine fungus, mangrove, salt adaptation

## Abstract

Mangrove sediments from New Caledonia were screened for xylanase sequences. One enzyme was selected and characterized both biochemically and for its industrial potential. Using a specific cDNA amplification method coupled with a MiSeq sequencing approach, the diversity of expressed genes encoding GH11 xylanases was investigated beneath *Avicenia marina* and *Rhizophora stylosa* trees during the wet and dry seasons and at two different sediment depths. GH11 xylanase diversity varied more according to tree species and season, than with respect to depth. One complete cDNA was selected (OFU29) and expressed in *Pichia pastoris*. The corresponding enzyme (called Xyn11-29) was biochemically characterized, revealing an optimal activity at 40–50 °C and at a pH of 5.5. Xyn11-29 was stable for 48 h at 35 °C, with a half-life of 1 h at 40 °C and in the pH range of 5.5–6. Xyn11-29 exhibited a high hydrolysis capacity on destarched wheat bran, with 40% and 16% of xylose and arabinose released after 24 h hydrolysis. Its activity on wheat straw was lower, with a release of 2.8% and 6.9% of xylose and arabinose, respectively. As the protein was isolated from mangrove sediments, the effect of sea salt on its activity was studied and discussed.

## 1. Introduction

The mangrove is a unique ecosystem located at an interface between terrestrial, estuarine, and near-shore marine ecosystems. It is exposed to tides and has to live with high concentrations of salts due to the evaporation of saline water. Mangrove trees have developed unique adaptations to survive high levels of salinity either by excreting salts through their leaves or by excluding them at the root surface with a filtration system. One of the main environmental factors controlling the distribution of vegetation appears to be sediment salinity, which is partly controlled by the position of the stand in the intertidal zone [1]. In addition, mangrove trees in areas with high salinity are usually characterized by a lower productivity and, thus, lower organic accumulation in their soils, modifying the sediment biogeochemistry. In this hostile environment, mangrove fungi contribute to the degradation of particulate organic matter into dissolved organic matter, including the lignocellulosic biomass [2]. In New Caledonia, mangrove zonation is typical of the semi-arid climate, with *Rhizophora stylosa* colonizing the lower intertidal zone and *Avicennia marina* growing at higher elevation with higher pore-water salinity and lower organic accumulation in sediments [3]. The fungi abundantly present within these mangroves constitute a very diverse ecological group (~100 to 1000 different taxa depending on the sample, many of them undescribed) characterized by a strong host specificity, the fungal communities associated with *A. marina* being very different from those associated with *R. stylosa* [4]. In a recent study, the prokaryotic and fungal communities in New Caledonian mangrove sediments were analyzed with respect to depth, vegetation cover, and season using a metabarcoding approach [5]. The prokaryotic community appeared to be exclusively shaped by sediment depth, while the fungal community was evenly distributed according to all the above criteria. In parallel, the functional diversity of the same New Caledonia mangrove sediments was examined by observing the distribution of fungal dye-decolorizing peroxidases (DyPs) as model lignin-modifying enzymes, thought to play a role in lignocellulose biomass degradation [6,7]. Using a functional metabarcoding approach, the diversity of expressed genes encoding fungal DyPs was investigated in surface and deeper sediments. The highest DyP diversity was observed for the surface layers beneath the *Rhizophora stylosa* area during the wet season. With regard to enzyme diversity, fungal adaptation to marine conditions, especially to high salt concentrations, is of great interest and was characterized for the mangrove fungus *Pestalotiopsis* sp. using a proteomic approach. This study revealed that the composition of secreted lignocellulolytic enzymes was strongly modified in the presence of sea salt, with increased secretion of xylanases and cellulases and lower production of oxidoreductases [8].

Xylanases (EC 3.2.1.8) are glycosidases that degrade xylans into xylooligosaccharide and xylose and thus help break down hemicellulose, one of the major components of plant cell walls [9]. Xylanases are classified in the CAZy database in GH families 5, 7, 8, 10, 11, and 43, with a predominance of GH10 and 11 [10]. Microbial xylanases are used in various industrial applications such as in the food industry (production of wine and beer, fruit juice, bread), for animal feed, biofuel production, and in the pulp and paper industry [9]. These industrial processes are conducted in a range of temperatures and pHs. In this context, xylanases were screened from marine environments, as they operate at different pH and temperature ranges from their terrestrial enzyme homologues and are also active in saline conditions [11]. Several studies have focused on xylanase activity screening to find new microbial activities and enzymes from seawater and mangrove forests, sediments, sponges and algae [11]. In these marine environments, fungal marine-derived xylanases were found to act at neutral to alkaline pH [9]. In saline conditions, xylanase XynMF13A from the mangrove fungus *Phoma* sp. MF13, heterologously produced in *Pichia pastoris,* was shown to have an optimal activity in 0.5 M NaCl and to keep 53% of its initial activity in 4 M NaCl [12].

In the present work, our objective was to evaluate the diversity of expressed genes encoding xylanases from mangrove sediments and to obtain the corresponding full-length cDNAs. From this data, one recombinant xylanase, Xyn11-29, was heterologously produced in *P. pastoris*, biochemically characterized, and its biochemical properties compared with those reported in the literature. This new xylanase was tested for the hydrolysis of lignocellulosic co-products, and its hydrolytic efficiency was compared with that of another xylanase, GH11.

## 2. Materials and Methods

### 2.1. Sediment Sampling, RNA Extraction and cDNA Synthesis

Sediment samples were collected from a mangrove wetland located in Saint Vincent Bay (21°55′58″ S, 166°4′30″ E) on the west coast of New Caledonia. As previously described [3], sediment samples were collected in three independent 10 m^2^ plots (A, B, C) located 50 m apart and defined in *Avicennia marina* (A) and *Rhizophora stylosa* (R) pristine areas. Three sediment cores (50 cm deep) were collected in 2016 at low tide with a stainless-steel corer (diameter 8 cm) in each 10 m^2^ plot during the wet (March) and dry (November) seasons. Oxic (0–10 cm deep) and anoxic (40–50 cm deep) fractions were collected from each core and a single composite sample per fraction and per plot (A, B or C) for each tree area (*A. marina* and *R. stylosa*) was prepared by mixing equal amounts of sediments. Per season (March or November), a total of 12 different composite samples were thus obtained and separately analyzed: (i) R1A, R1B, R1C and R2A, R2B, R2C corresponding, respectively, to the oxic and anoxic fractions from the three plots (A, B, C) designated in the *R. stylosa* area (R) and (ii) A1A, A1B, A1C and A2A, A2B, A2C corresponding, respectively, to oxic and anoxic fractions of the three plots (A, B or C) localized in the *A. marina* area (A). All composite samples were frozen and kept at −70 °C until use.

Total RNA was extracted from 8–12 g of each composite sample using the RNeasy PowerSoil Total RNA kit, as previously described in Ben-Ayed et al. [6]. After a quality and quantity check, cDNA synthesis was conducted on 500 ng of total RNA using the Mint-2 cDNA synthesis kit (Evrogen, Moscow, Russia). Resulting cDNAs were then purified and used as templates to specifically capture full-length transcripts encoding fungal GH11 xylanases as previously described [13].

### 2.2. High-Throughput Sequencing of Fungal GH11 Expressed Genes and Full-Length Xyn11-29 Sequence Retrieval

Captured cDNAs from each sediment sample (12 per season) were first used as templates to specifically amplify fragments of expressed fungal GH11 xylanase-encoding genes using tagged degenerate primers fungGH11-F (5′-Tag-GGVAAGGGITGGAAYCCNGG-3′) and fungGH11-R (5′-Tag-TGKCGRACIGACCARTAYTG-3′) (Supplementary Table S1, [14]. All PCR amplifications were performed in triplicate in 25 μL reaction mixtures containing 2.5 μL of 10X buffer (Invitrogen, ThermoFisher Scientific, Waltham, MA, USA), 0.75 μL of MgCl_2_ (50 mM), 2.5 μL of dNTPs (2 mM each), 1 μL of each primer (20 μM, Invitrogen), 0.3 μL of bovine serum albumin (20 mg mL^−1^), 0.1 μL of Taq DNA polymerase (5U μL^−1^, Invitrogen), and 20 ng of cDNA. The cycling conditions were 3 min at 94 °C followed by 35 cycles (45 s at 94 °C, 45 s at 50 °C and 45 s at 72 °C) and 10 min at 72 °C. Control reactions without nucleic acid were systematically run in parallel. Amplicons from the three independent PCR reactions were pooled and purified using the Agencourt AMPure XP Kit (Beckman Coulter Diagnostics, Brea, CA, USA) and quantified by fluorometry using a Qubit 2.0 fluorimeter and the Qubit dsDNA H assay kit (Invitrogen). Per season (March or November), an equimolar mix of the tagged PCR products obtained for the 12 different sediment samples was prepared and sequenced by FASTERIS (FASTERIS, Plan-les-Ouates, Switzerland) on an Illumina MiSeq sequencer (2 × 250 bp).

In parallel, a plasmid library containing full-length GH11 cDNAs captured from the 24 sediment samples was constructed as previously described [13], using the pDR196 *E. coli*/*S. cerevisiae* shuttle expression vector. The full-length cDNA sequence encoding the Xyn11-29 was retrieved from this plasmid library by PCR using two primers pairs (PMA1: 5′-CTCTCTTTTATACACACATTC-3′/Xyn11-29-F: 5′-TCCGTCAGCTGCAACGGAGCG-3 and ADH: CATAAATCATAAGAAATTCGC/Xyn11-29-R: 5′-CGCTCCGTTGCAGCTGACGGA-3′) specifically targeting both plasmid and OFU29 sequences.

### 2.3. Bioinformatic Analysis and Statistics

As described in Ben-Ayed et al. [6], we used the pipeline Frogs to analyze Miseq sequences. Briefly, paired-end sequences were merged and demultiplexed to perform denoising procedures (presenting no ambiguous bases and in the range 230–350 bp). Trimmed sequences were clustered using Swar. Chimeras and scarce sequences (less than 0.005% of total abundance) were discarded. A normalization threshold of 10,311 sequences was applied to compare samples. The effects of environmental factors (tree, season, and depth) on the composition of expressed fungal genes encoding GH11 xylanases were tested using non-parametric permutation-based multi-variate analysis of variance (PERMANOVA, adonis function; [15] based on abundance dissimilarity (Bray–Curtis) matrices). This analysis was performed using the VEGAN package (http://cran.r-project.org, accessed on 19 April 2021) in R. The sequence data generated in this study were deposited in the EMBL-ENA public database (PRJEB43346 for the first sampling campaign (March) dataset and PRJEB43343 for the second sampling campaign (November) dataset).

The ProtParam tool (http://web.expasy.org/protparam/, accessed on 5 May 2021) was used to predict the theoretical molecular mass, and molar extinction coefficient of Xyn11-29. The signal peptide sequence was predicted using the tool available at http://www.cbs.dtu.dk/services/SignalP-4.1/ (accessed on 5 May 2021). *N*-Glycosylation sites were predicted via the NetNGlyc 1.0 server (http://www.cbs.dtu.dk/services/NetNGlyc/, accessed on 5 May 2021). For sequence comparison, BlastP was used to search for sequences with similarity to Xyn11-29 at NCBI portal (https://blast.ncbi.nlm.nih.gov/Blast.cgi, accessed on 5 May 2021) using the non-redundant protein sequence data. For the phylogenetic analysis, Muscle alignment was used at MEGA X (https://www.megasoftware.net/, accessed on 5 May 2021). The evolutionary history was inferred by using the maximum likelihood method and JTT matrix-based model under MEGA X [16,17]. The tree with the highest log likelihood (−5562.80) is shown. The percentage of trees in which the associated taxa clustered together is shown next to the branches. Initial trees for the heuristic search were obtained automatically by applying Neighbor-Join and BioNJ algorithms to a matrix of pairwise distances estimated using the JTT model and then selecting the topology with the highest log likelihood value. The tree is drawn to scale, with branch lengths measured in the number of substitutions per site. This analysis involved 51 amino acid sequences. There were, in all, 218 positions in the final dataset.

### 2.4. Strains for Cloning and Heterologous Expression

*Escherichia coli* strain DH5α (Promega, Charbonnières, France) was used for vector storage and propagation. *P. pastoris* strain X33 (Invitrogen) was used for the heterologous expression of the Xyn11-29 encoding synthetic gene after the optimization of codons (GenScript, Piscataway, NJ, USA).

### 2.5. Cloning of the Xyn11-29 cDNA in Pichia Pastoris and Screening of Transformants

Xyn11-29 was produced using the 3PE Platform (*P. pastoris* Protein Express: www.platform3pe.com/, accessed on 5 May 2021). The cDNA encoding Xyn11-29 was synthesized after codon optimization for *P. pastoris* (GeneArt, Regensburg, Germany) and inserted into the vector pPICZαA (Invitrogen) using *Xho*I and *Xba*I restriction sites in frame with the α-factor and the (His)6 tag at the C terminus of the recombinant protein.

The *Pme*I-linearized pPICZαA recombinant plasmid was inserted into *P. pastoris*-competent cells by electroporation. Zeocin-resistant transformants were then screened for protein production. *P. pastoris* strain X33 and the pPICZαA vector are components of the *P. pastoris* Easy Select Expression System (Invitrogen). Electrocompetent cell preparation, electroporation, and screening were carried out as previously described [18]. Protein production was confirmed by SDS-PAGE.

### 2.6. Production and Purification of Recombinant Xyn11-29

The best producing transformant was grown in 2 L of BMGY (10 g L^−1^ glycerol, 10 g L^−1^ yeast extract, 20 g L^−1^ peptone, 3.4 g L^−1^ YNB, 10 g L^−1^ ammonium sulfate, 100 mM phosphate buffer pH 6 and 0.2 g L^−1^ of biotin) in flasks shaken at 30 °C in an orbital shaker (200 g) for 16 h to an OD600 of 2–6. Cells were then pelleted by centrifuging at 3500× *g* for 5 min and resuspended in 100 mL of a BMMY medium (10 g L^−1^ yeast extract, 20 g L^−1^ peptone, 3.4 g L^−1^ YNB, 10 g L^−1^ ammonium sulfate, 100 mM phosphate buffer pH 6 and 0.2 g L^−1^ of biotin). Xyn11-29 production was induced at 20 °C in an orbital shaker (200 g) by adding 3% (*v*/*v*) methanol daily for three days.

The supernatant (500 mL) was collected after harvesting cells by centrifugation at 3500× *g* for 5 min at 4 °C. After adjusting the pH to 7.8, the supernatant was filtered on 0.45 µm filters (Millipore, Molsheim, France) and loaded onto a 5 mL HisTrap HP column (GE healthcare, Buc, France) connected to an Akta Xpress system (GE healthcare). Prior to loading, the column was equilibrated with buffer A Tris-HCl 50 mM pH 7.8, NaCl 150 mM and imidazole 10 mM. The (His)6-tagged recombinant enzyme was eluted with buffer B Tris-HCl 50 mM pH 7.8, NaCl 150 mM and imidazole 500 mM. The fractions eluted containing the purified protein were pooled, concentrated with a 10 kDa Vivaspin concentrator unit (Sartorius, Palaiseau, France) and dialyzed against 50 mM sodium acetate buffer pH 5.2.

Protein concentration was determined using a Nanodrop ND-2000 spectrophotometer (Thermo Fisher Scientific, Waltham, MA, USA) by adsorption at 280 nm with theoretical molecular masses (22,807 Da) and molar extinction coefficients (66,935 M^−1^ cm^−1^) calculated from protein sequence using Expasy tools. A fraction of eluate was loaded onto 10% Tris-glycine precast SDS-PAGE (Bio-Rad, Marnes-la-Coquette, France) to check protein purity and integrity. The molecular mass under denaturing conditions was determined with a PageRuler Prestained Protein Ladder (Thermo Fisher Scientific).

### 2.7. Standard Conditions of Xylanase Activity

Xylanase activities were measured by the quantification of reducing sugar release during incubation in the presence of beechwood xylan (Roth, Karlsruhe, Germany) (0.5%, *w*/*v*) in 50 mM citrate phosphate buffer at pH 5.5 and a temperature of 40 °C. Reducing sugars were quantified using the ferricyanide-based method described by Kidby and Davidson [19]. Measurements were performed in triplicate. 

### 2.8. Influence of Temperature and pH on Xyn11-29 Activity and Enzyme Stability

To determine the influence of temperature and pH, the purified enzyme at 0.0541 mM was used. To determine the optimal pH, Xyn11-29 activity was quantified in the presence of beechwood xylans in a 50 mM sodium tartrate buffer from pH 2–4 and citrate-phosphate buffer from pH 5 to 7 at 30 °C. For the temperature profiles, the recombinant xylanase was incubated over the temperature range 20–70 °C in the presence of beechwood xylans in 50 mM citrate-phosphate buffer at pH 5.5.

To analyze thermal stability, Xyn11-29 was incubated at different temperatures (35, 40 and 50 °C) for 48 h in 50 mM citrate-phosphate buffer at pH 5.5, with regular sampling. For pH stability, the recombinant enzyme was incubated in 50 mM citrate-phosphate buffer at pH 5; 5.5 and 6 at 35 °C for 48 h. After incubation, activity was measured in standard conditions in the presence of beechwood xylans.

### 2.9. Influence of Sea Salt on Xylanase Activity

To determine the effect of sea salt on enzyme activity, activity was measured in standard conditions after adding sea salt (1–5% wt/vol) in 50 mM citrate-phosphate buffer at pH 5.5. To compare the effect of sea salt, a similar experiment was also performed with the purified GH11 xylanase *Tx*-Xyn11 from the bacterium *Thermobacillus xylanilyticus* in 50 mM sodium acetate buffer at 60 °C and at pH 5.8 [20].

### 2.10. Kinetic Properties

Kinetic parameters were determined in standard conditions with beechwood as substrate at concentrations of 1, 2, 5, 8, and 10 g/L. Experiments were performed in triplicate. Lineweaver–Burk plots were used to calculate the Michaelis constant *K*_M_ and the enzyme turnover value *k*_cat_. The normalized equation *v* = (*k*_cat_/*K*_M_)[S]/(1 + [S]/*K*_M_) yielded the catalytic efficiency values (*k*_cat_/*K*_M_) with their corresponding standard errors.

### 2.11. Lignocellulosic Biomass Hydrolysis

For the study of xylanase hydrolysis capacity, destarched wheat bran and wheat straw (Apache variety, 2 mm) provided by Agro Industrie Recherches Developpement (ARD, Pomacle, France) were used. Lignocellulose substrates (500 mg) were hydrated for 1 h in 10 mL of 50 mM citrate-phosphate buffer at pH 5.5 at 35 °C and in 10 mL of 50 mM sodium acetate buffer pH 5.8 at 60 °C for hydrolysis with the Xyn11-29 and *Tx*-Xyn11 experiment, respectively. Xyn11-29 and *Tx*-Xyn11 were then added at 100 IU per gram of substrate. Experiments were performed in duplicate under constant magnetic stirring (350 rpm) for 48 h. Hydrolysate samplings were performed after 0, 6, 24, and 48 h of incubation and the reaction was stopped by boiling at 100 °C for 10 min. Centrifugation (3000× *g*, 10 min) separated supernatant and residual biomass. The activity of *Tx*-Xyn11 was also determined to compare the hydrolytic efficiency of the xylanase Xyn11-29. *Tx*-Xyn11 was selected for its high hydrolytic potential for lignocellulosic biomass [21].

The identification and quantification of the sugars released were carried out by HPAEC-PAD using a CarboPac PA-1 anion exchange column (4 × 250 mm, Dionex, Thermo Fisher Scientific, Waltham, MA, USA). All samples were hydrolyzed with 1M H_2_SO_4_ for 1 h at 100 °C. l-Fucose was used as internal standard and was added to the samples at a final concentration of 100 µg mL^−1^. Each sample was then filtered (0.22 µm) before injection into the column. l-Arabinose and d-xylose were used as standards.

## 3. Results

### 3.1. Diversity of Fungal Expressed Genes Encoding GH11 Xylanases

**Figure 1:**
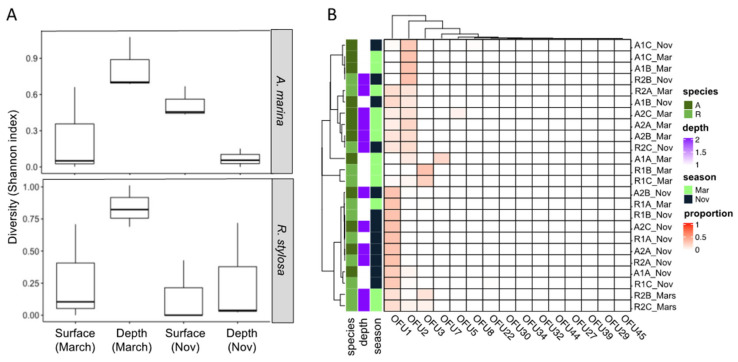
Diversity of expressed genes encoding xylanases in surface and deeper sediments collected beneath two tree species (*Avicennia marina* and *Rhizophora stylosa*) during the wet (March) and dry (November) seasons. (**A**) Diversity estimated with the Shannon index and (**B**) distribution of the different operational functional units (OFUs).

The diversity of expressed genes encoding fungal GH11 xylanases was investigated in surface and deeper mangrove sediments beneath *A. marina* (A) and *R. stylosa* (R) trees during the wet (March) and dry (November) seasons in three independent plots. This diversity analysis was performed using a classical functional metabarcoding approach based on cDNAs previously enriched in fungal GH11 sequences. The normalized GH11 xylanase dataset consisted of 247,464 sequences distributed among only 15 different operational functional units (OFUs) corresponding to 15 putative GH11 xylanase encoding cDNAs (Figure 1, Supplementary Table S2). The number of OFUs per sediment sample ranged from one to seven 7. GH11 diversity, estimated with the Shannon index, was systematically higher in deep layers during the wet season (Figure 1A, Supplementary Table S3). The highest GH11 diversity was observed for the deep layers beneath the *R. stylosa* area during the wet season. By contrast, the lowest GH11 diversity was observed in the deep layers beneath the *A. marina* area during the dry season (Figure 1A, Supplementary Table S3).

Two OFUs (OFU1 and OFU2) largely dominated the GH11 expressed genes, representing 52% and 33% of the total number of sequences, respectively. Regarding their distribution, the OFUs were detected in almost all sediment samples independently of the season. However, OFU2 seemed more strongly expressed during the wet season (March) than the dry one (November) and not in all sediment samples (Figure 1B).

The difference in the composition of expressed GH11 xylanase encoding genes assessed through a non-parametric multivariate analysis of variance (PERMANOVA) highlighted strong effects of the season (*p* = 0.01, *R*^2^ = 0.158) and of the tree species (*p* = 0.02, *R*^2^ = 0.104). The sediment depth had a lower impact (*p* = 0.20, *R*^2^ = 0.039) on the composition of the expressed fungal genes (Table 1).

### 3.2. Phylogeny of the Xylanase Sequences

**Figure 2:**
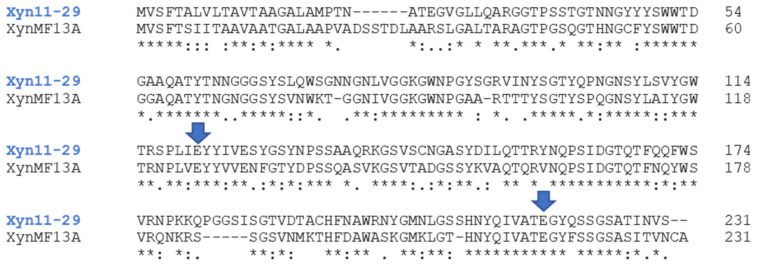
Sequence alignment of Xyn11-29 with the biochemically characterized xylanase XynMF13A isolated the mangrove fungus *Phoma* sp. (GenBank accession number AVV62005.1). The highly conserved glutamate catalytic residues are marked with blue arrows. Alignment was produced with Clustal Omega, and symbols below the sequences indicate full conservation of the same (asterisk) or equivalent residues (colon) and partial residue conservation (dot).

The selection criteria for the enzyme target were: (i) representativity of the OFU in the mangrove sediments; (ii) obtainment of the full-length coding sequence by PCR; and (iii) production of the recombinant xylanase in *P. pastoris*. From the most abundant OFUs, OFU 2 and OFU 5 were amplified in our standard protocol, but not OFU1. The two amplified OFUs were not produced in *P. pastoris*, so we decided to study OFU29, a sequence close to OFU1 (99% identity 80/81 amino acid identity) that could be produced in the heterologous host strain. A BlastP was conducted at NCBI (non-redundant protein sequences) using Xyn11-29 sequence as a query. Xylanase sequences of the family GH11 from the orders Agaricales and Sebacinales emerged as proteins with the highest amino acid sequence identity (here, give the range of % identity for the first 10 best hits) (Table 2). Xyn11-29 underwent a phylogenetic analysis with the 50 best hit sequences obtained from the BlastP analysis. However, the origin of the sequence could not be inferred by this approach (not shown), probably owing to strong conservation of xylanase amino acid sequences among Agaricomycetes. In the current state of the available data, we can only suggest that Xyn11-29 has a fungal origin and belongs to a basidiomycete of the Agaricomycetes class. Alignment with the biochemically characterized mangrove-derived xylanase XynMF13A confirmed that Xyn11-29 contained the two highly conserved catalytic residues characteristic of GH11 xylanases (Glu120 and Glu217) (Figure 2) and an analysis of the domain architecture showed that the 231 amino acid sequence was composed of a catalytic domain without a carbohydrate binding module.

### 3.3. Heterologous Production and Purification of the Recombinant Xyn11-29

After the transformation of *P. pastoris* with the recombinant vector pPICZα-A containing the Xyn11-29 encoding cDNA, 50 transformants were selected for their resistance to zeocin and were then screened for the presence of Xyn11-29 in the extracellular medium. The best transformant was selected based on the band intensity corresponding to the recombinant protein and visualized following SDS-PAGE. The expected molecular weight for Xyn11-29 was 22.8 kDa, and after purification, the protein ran on SDS-PAGE at approximately 20 kDa with bands of slightly higher molecular masses probably corresponding to hyperglycosylated isoforms as already observed (Supplementary Figure S1) [6]. Xyn11-29 was predicted to possess three potential *N*-glycosylations at positions 4, 77, and 113 (starting from the first amino acids of the mature protein M PTNATEGVGL), as predicted via the NetNGlyc 1.0 Server (http://www.cbs.dtu.dk/services/NetNGlyc/, accessed on 5 May 2021). The recombinant protein was purified by affinity chromatography using an IMAC column, yielding a protein concentration of 1.33 mg mL^−1^, with a total activity of 1170 IU mL^−1^ (Figure S1). The specific activity of the Xyn11-29 is 877 IU mg^−1^.

### 3.4. Effect of pH and Temperature on the Activity and Stability of Xyn11-29

**Figure 3:**
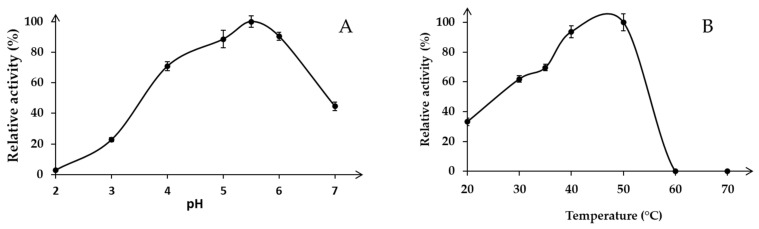
Effect of pH and temperature on enzyme activity: (**A**) Optimal pH in 50 mM sodium tartrate buffer from pH 2 to 4 and citrate-phosphate buffer from pH 5 to 7 at 30 °C; (**B**) Optimal temperature for beechwood xylan hydrolysis (0.5%, *w*/*v*) in the range 20–70 °C at pH 5.5. Activity values were calculated as a percentage of maximum activity (set to 100%) at optimum temperature and pH. Each data point (mean +/− standard deviation) is the result of triplicate experiments.

Recombinant Xyn11-29 was active under acidic pH conditions with an optimum at pH 5.5 for beechwood xylan hydrolysis in the pH range tested (Figure 3A). At lower pH, the Xyn11-29 activity markedly decreased, especially between pH 2 and 4, and almost no activity was detected at pH 2. At a higher pH, activities significantly decreased to less than 50% at pH 7. In the tested temperature range, the activity was optimal between 40 °C and 50 °C, while the enzyme was less active at lower temperatures (30–70% of its activity found at 50 °C) (Figure 3B). We note that Xyn11-29 was not active at temperatures above 60 °C. In conclusion, the optimal activity of Xyn11-29 was obtained at 40–50 °C in a 50 mM citrate-phosphate buffer at pH 5.5.

**Figure 4:**
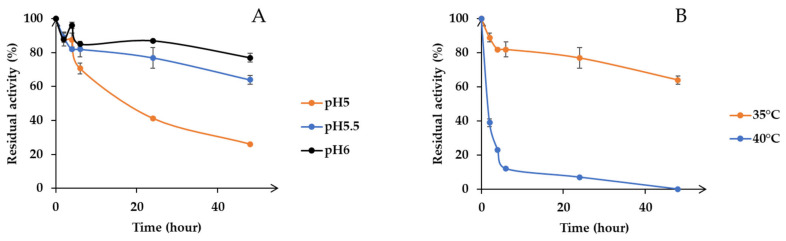
pH and temperature stability. (**A**) pH stability in 50 mM citrate-phosphate buffer from pH 5 to 6 at 35 °C; (**B**) Temperature stability from 35 °C to 50 °C at pH 5.5. Activity values were calculated as a percentage of maximum activity (set to 100%) at optimum temperature and pH. Each data point (mean +/− standard deviation) is the result of triplicate experiments.

Secondly, pH stability of the enzyme was determined. Activities were estimated after incubation at various pH values (5, 5.5 and 6) (Figure 4A). Results show that Xyn11-29 activity was relatively stable at pH 6, exhibiting 80% of its maximal activity after 48 h incubation. By contrast, at pH 5.5, the recombinant xylanase retained only 64% of its initial activity after 48 h of incubation, and at pH 5, 60% of its activity was lost after only 24 h of incubation (Figure 4A). Xyn11-29 displayed a relatively high thermal stability at 35 °C, with a half-life longer than 48 h (Figure 4B). Half-lives decreased with temperature, 1 h at 40 °C and only 3 min at 50 °C (data not presented on the graph for 50 °C). Taking into account the temperature and pH stabilities of Xyn11-29, standard conditions (40 °C, pH 5.5) were used for determining kinetic properties, and lignocellulosic biomass hydrolyses were performed at 35 °C and pH 5.5 for 48 h. 

### 3.5. Catalytic Properties

The enzyme exhibited activity against beechwood xylan, with a *K*_M_ value of 3.3 g L^−1^ and a catalytic efficiency *(k*_cat_/*K*_M_) of 206 g L^−1^ s^−1^ (Table 3). Values measured for the fungal mangrove-derived xylanase, XynMF13A [12], were close to that found for Xyn11-29, with *K*_M_ and *k*_cat_/*K*_M_ values of 3.16 g L^−1^ and 340 g L^−1^ s^−1^, respectively, using beechwood xylan as the substrate.

### 3.6. Xylanase Activity against Lignocellulosic Biomass

**Figure 5:**
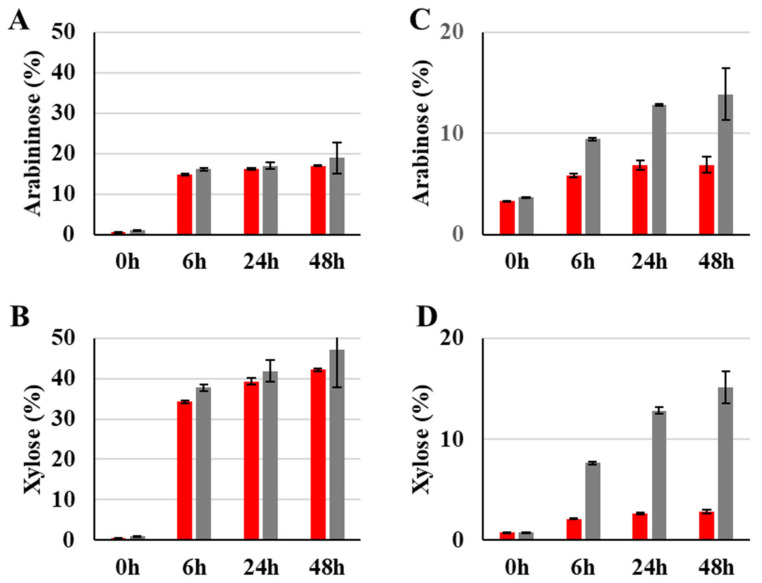
Enzymatic hydrolysis capacity on destarched wheat bran (**A**,**B**) and wheat straw (**C**,**D**) of Xyn11-29 xylanase (red bars) compared with *T. xylanolyticus* xylanase, *Tx*-Xyn11 (gray bars) after 0, 6, 24, and 48 h incubation at pH 5.5 at 35 °C and at pH 5.8 at 60°C for Xyn11-29 and Tx-Xyn11, respectively. Results are expressed as a percentage of released sugars (arabinose and xylose). Each data point (mean +/− standard deviation) is the result of duplicate experiments.

Xyn11-29 xylanase activity was compared with the xylanase from *Thermobacillus xylanilyticus* (*Tx*-Xyn11), already described for its efficiency for wheat straw xylan fractionation [21]. Xyn11-29 exhibited a high hydrolysis capacity on destarched wheat bran, with 40% and 16% of xylose and arabinose released after 24 h of hydrolysis, respectively (Figure 5A,B). These values are very similar to those obtained with *Tx*-Xyn11 xylanase. Conversely, the efficiency of the Xyn11-29 xylanase was low on wheat straw, with 2.6% and 6.9% of xylose and arabinose released after 24 h, respectively, while the efficiency of the *Tx*-Xyn11 xylanase was in general higher (Figure 5C,D).

### 3.7. Influence of Sea Salt on Xylanase Activity

**Figure 6:**
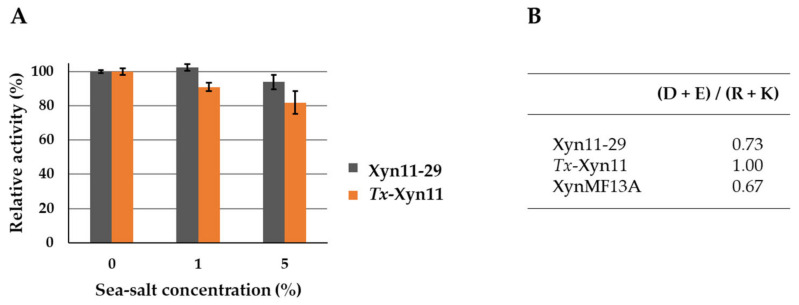
Sea salt effect on Xyn11-29 xylanase activity and ratios of negatively to positively charged amino acids for Xyn11-29, *T. xylanolyticus*, *Tx*-Xyn11, and *Phoma* sp. xylanase, XynMF13A. (**A**) Sea salt was used at concentrations of 1% and 5%. Activities are expressed as a percentage of the activity without sea salt (set to 100%). Each data point (mean +/− standard deviation) is the result of triplicate experiments. (**B**) Negatively charged (D + E) to positively charged (R + K) amino acid ratios.

The influence of sea salt on xylanase activity was analyzed by activity measurement in standard conditions (40 °C at pH 5.5) with 1% and 5% of salt on the buffer used (Figure 6A). In the tested conditions, sea salt had no significant effect on Xyn11-29 xylanase activity even at 5% (wt/vol). In comparison, *Tx*-Xyn11 showed a slight decrease in its activity, particularly at 5% sea salt, with a reduction of 20% of its initial activity. In addition, analysis of the enzyme primary structure showed that Xyn11-29 had a very low recurrence of negative surface charges, with (D + E)/(R + K) = 0.73, similar to what was calculated for XynMF13a (Figure 6B). The terrestrial *Tx*-Xyn11 had an even balance of negative and positive charges.

## 4. Discussion

The functional diversity of fungal xylanases was investigated during both wet and dry seasons in New Caledonian mangrove sediments, from the surface to deeper horizons under the two most common tree species in this region (*Avicennia marina* and *Rhizophora stylosa*). Xylanases are involved in breaking down hemicellulose, one of the major components of plant cell walls, and they are largely characterized in the fungal kingdom. They were accordingly selected as markers for the biomass enzymatic degradation in the mangrove soil [7]. Compared with soils from terrestrial forests, which can harbor up to 103 GH11 OFUs [14], with only 15 GH11 OFUs, mangrove sediments showed a much lower diversity. This lower diversity observed may be the result of physical and chemical gradients associated with mangrove sediments that are inimical to fungal colonization and related enzymatic activities (e.g., low oxygen and nitrogen contents) [22]. The diversity of fungal communities was investigated in a previous study and as observed here for the expressed GH11 genes, the fungal diversity was also strongly affected by season and tree species [5]. This impact of tree species on fungal communities and their expressed genes is consistent with previous work conducted in classical forest ecosystems demonstrating that tree species select diverse soil fungal communities expressing different sets of lignocellulolytic enzyme-encoding genes [14]. Interestingly, the diversity of expressed genes encoding GH11 xylanases was systematically higher in deep layers during the wet season. Such higher expressed gene diversity may result from the combination of optimal condition for fungal xylanase activities such as higher carbon content in deeper layers [22] and a wet season favorable to fungal colonization in mangrove ecosystems [23].

Xyn11-29 (OFU29) was selected from the microbial diversity of the New Caledonia sediments as it was the closest homolog to the most abundant OFU (OFU1) with 99% identity, and OFU1 could not be produced in *P. pastoris*. In the current state of the data, the origin of the protein could not be inferred by a phylogenetic approach. However, we make the supposition that the protein is a xylanase from the family GH11, which we call Xyn11-29. In addition, Xyn11-29 shares high similarity with GH11 enzymes from the Agaricomycetes class, within the Agaricomycotina sub-phylum. In a previous study, the same mangrove sediments showed a predominance of Ascomycota (76.8–94.5% of total ITS) and Basidiomycota (5.3–18.3% of total ITS) [3]. In addition, the most abundant OFU identified in the same mangrove sediments using DyP as an alternative enzymatic marker was attributed to a fungus belonging to the Agaricomycetes class, in agreement with our present results [6].

A characterization of the mangrove-derived Xyn11-29 was conducted after its production in *P. pastoris* and its purification. Concerning its general properties, Xyn11-29 was shown to possess only a catalytic module without CBM, as is the case for 80% of xylanases (family GH11) from eukaryotic organisms [24]. Xyn11-29 has the average size for a xylanase of around 20 kDa and possesses the two key catalytic residues characteristic of the xylanases (Glu120 and Glu217) [25]. Kinetic parameters were determined and compared with the biochemically characterized XynMF13A isolated from the Shankou mangrove in Guangxi province, China [12]. These two mangrove-derived xylanases exhibited very close kinetic parameters, with a *K*_M_ of 3 g L^−1^ against beechwood xylans and a catalytic efficiency (*k*_cat_/*K*_M_) around 200–300 g L^−1^ s^−1^. No other kinetic values could be found in the literature concerning other marine-derived xylanases. Compared with terrestrial xylanases, *K*_M_ values were relatively close, with values of 3.42–9.96 g L^−1^ for xylanases from *Aspergillus oryzae* and *Phanerochaete chrysosporium* (for a review see [26]). Concerning stability and activity as a function of temperature and pH, Xyn11-29 exhibited rather generic properties, with an optimal activity at pH 5–6, and a maximum activity between 40 °C and 50 °C. In addition, the recombinant protein was stable at pH 5 and 6, with a low stability at high temperature, especially at 50 °C, with a half-life of 3 min. However, Xyn11-29 possesses high thermal stability at lower temperature and so can be used at moderate temperature. The mangrove-derived XynMF13A has similar properties [12], and among the multiple examples of fungal xylanases found in terrestrial fungi the same properties could be mostly found [27,28,29,30]. Considering these properties, we can conclude that Xyn11-29 shares the general characteristics of the xylanases found in terrestrial fungi and with the sole characterized purified xylanase isolated from a marine area, namely mangrove sediments.

As xylanases have already been gainfully used for several years and, more recently, in lignocellulose degradation. In the context of plant biomass refining [31], we tested and compared Xyn11-29 with xylanase *Tx*-Xyn11 from *T. xylanilyticus*, known to efficiently hydrolyze lignocellulosic biomass [21,32]. Xyn11-29 exhibited similar efficiency to Tx-xyn11 for the release of sugars (xylose and arabinose) from destarched wheat bran. The release was high, reaching 17% and 42%, respectively, for arabinose and xylose after 48 h. Lower xylose and arabinose release was obtained on wheat straw for Xyn11-29 with 2.8% and 6.9%, respectively. The efficiency of Tx-xyn11 on wheat straw was higher. The high hydrolytic efficiency of Xyn11-29 on destarched wheat bran compared with wheat straw could be explained by the low recalcitrance of wheat bran relative to wheat straw, as the lignin content in wheat bran is low (less than 5% dry matter). The lower ability of Xyn11-29 to hydrolyze the xylans from wheat straw relative to the xylanase Tx-xyn11 could be explained by the kinetic parameters of both enzymes on xylans with a low degree of branching, as is the case for wheat straw xylans [33]. When measured on beechwood xylans, the catalytic efficiency of Xyn11-29 reached 216 L g^−1 ^s^−1^, whereas the *k*_cat_/*K*_M_ was 4684 L g^−1^ s^−1^ for Tx-xyn11. However, this ability to degrade lignocellulosic substrates to some extent and at moderate temperature suggests biotechnological applications for which Xyn11-29 could be usefully tested, such as in the pulp and paper industry and the food sector.

Several studies have been undertaken on marine microorganisms in order to discover new biocatalysts with activity resistant to saline conditions and, more generally, to harsh conditions (as required in the industry). For instance, the dye-colorizing sector and the food industry need more robust biocatalysts that could exhibit these properties, and marine organisms that have adapted to salty environments are thought to produce and secrete enzymes that are active, especially in saline conditions [34]. Xyn 11-29 is active at up to 5% of sea salt. Activities of the mangrove-derived XynMF13A were tested in the presence of NaCl and were quite stable after 1 h incubation in 3 M NaCl [12]. In addition, this xylanase retained 80% of its initial activity in up to 4 M NaCl. Research has been seeking to determine why some of these marine enzymes and not others possess this interesting property of acting in saline conditions. To our knowledge, only three other studies with the objective of screening marine-derived fungal strains for xylanase activities have been published [35,36,37]. In these studies, only the crude extracellular medium of the fungal culture was characterized and not the purified xylanase, so no data were provided on the effect of salt on the related enzymes. However, other studies are available on the effect of the saline conditions on the activity of other enzymes. For instance, the dye-decolorizing peroxidase DyP1, purified from a fungal strain of the same New Caledonia mangrove, was negatively affected by sea salt, with a complete inhibition of its activity at 3% of sea salt. There was a similar effect on the terrestrial *T. versicolor* DyP1, *Tv*DyP1 [6]. Likewise, but with a less pronounced effect, a laccase purified from the marine-derived *Stemphylium lucomagnoense* strain from the Tunisian coast lost 50% of its initial activity in 4% of sea salt [38]. By contrast, two laccases obtained from *Pestalotiopsis* sp. KF079 isolated from the Baltic sea mudflats were positively activated to 200–360% of their initial activities [34]. Some studies showed that salt-adapted enzymes were characterized by highly negative surface charges thought to contribute to protein stability and activity in extreme osmotic conditions [39,40,41]. In addition, solvation has been suggested to be a key requirement for maintaining solubility and activity of enzymes in low water activity [42]. In this context, hydrogen bonds between negatively charged side chains and water molecules become critical to maintaining a stable hydration shell surrounding the enzyme. In line with this hypothesis, the laccase *Ps*Lac2 of *Pestalotiopsis* sp. KF079 was shown to possess a high ratio of negative to positive amino acids (D + E)/(R + K) of 3.95. Other enzymes with an activity that remained stable or decreased in the presence of sea salts, including Xyn11-29, have a appreciably lower ratio with values in the range from 0.67 (mangrove-derived XynMF13A) to 1.0 (terrestrial-derived *Tx*-Xyn11). Also, the mangrove-derived DyP1, which was negatively affected by sea salt, presents a balanced ratio of 1.19, similar to that of *Tx*-Xyn11. Accordingly, and as was concluded for mangrove-derived DyP1, we hypothesized that these two enzymes were proteins reminiscent of those of terrestrial origin in their behavior in saline conditions. A recent study has shown that leaves and aerial bodies predominantly contain fungi derived from well-studied terrestrial environments with functions similar to their terrestrial plant counterparts [43]. By contrast, sediments and pneumatophores are colonized by marine fungi, especially since they are periodically submerged by tides, requiring them to grow in an environment that experiences large and rapid fluctuations in salinity. Further research will be needed to extend our work on the mangrove by cloning other functional enzymes and studying their origin to gain more general insight into this complex marine environment. Through a broader inventory of functional enzymes, a systematic characterization of model enzymes such as DyP and xylanase should yield interesting data on the marine versus terrestrial origin of the producing strains.

## 5. Conclusions

Mangroves are original, environmentally essential ecosystem located at the intertidal zone between land and sea. They are adapted to a gradient of salinity, temperature, humidity, and tree species, resulting in a diversity of the microbiota and enzymes they produce. In this context, we have developed an approach to study the functional diversity of the New Caledonia mangrove, which could find use in biotechnological applications. The selected enzyme model, Xyn11-29, was shown to possess the general characteristics of xylanases from family GH11, and to recall terrestrial xylanases with biotechnological abilities like those of their terrestrial homologs.

## Figures and Tables

**Table 1:** Effect of environmental factors on the composition of expressed fungal genes encoding GH 11 xylanases.

	Df	*F*	*p*	*R* ^2^
**Variable**				
Tree species	1	4.369	0.02	0.104
Sediment depth	1	1.629	0.20	0.039
Season	1	6.641	0.01	0.158
**Interaction**				
Tree × Depth	1	5.376	0.01	0.128
Tree × Season	1	4.755	0.02	0.113
Depth × Season	1	1.674	0.18	0.040
Tree × Depth × Season	1	1.579	0.19	0.038
**Residuals**	16			

The differences between groups were tested using PERMANOVA analysis on Bray–Curtis dissimilarity matrices. Abbreviations: Df, degrees of freedom; *F*, *F*-test statistic.

**Table 2:** Putative xylanases presenting the highest amino acid identities with Xyn11-29. The sequences (GenBank accession number) and the fungal species are listed with their corresponding order in brackets, together with the percentage of identity and the number of residue pairs considered for each comparison.

	Xyn11-29
KAF9530414.1 *Crepidotus variabilis* (Agaricales)ćKAF9459224.1 *Lepista nuda* (Agaricales) PVF97526.1 *Serendipita vermifera* ‘subsp. *bescii*’ (Sebacinales) ESK83196.1 *Moniliophthora roreri* (Agaricales) KAF9530413.1 *Crepidotus variabilis* (Agaricales) PVF91255.1 *Serendipita vermifera* ‘subsp. *bescii*’ (Sebacinales) KAF5345258.1 *Leucoagaricus leucothites* (Agaricales) CDS82539.1 uncultured eukaryote KTB33854.1 *Moniliophthora roreri* (Agaricales) KIM21487.1 *Sebacina vermifera* (Sebacinales)	195/231(84%) 193/231(84%) 195/235(83%) 187/231(81%) 189/231(82%) 188/233(81%) 193/232(83%) 188/233(81%) 182/231(79%) 182/231(79%)

**Table 3:** Kinetic constants of the recombinant Xyn11-29 produced in *P. pastoris*. Experiments were performed in triplicate against beechwood at various concentrations in the range 1–10 g L^−1^. Activities were measured at 40 °C pH 5.5.

	Specific Activity (IU mg^−1^)	*K*_M_ (g L^−1^)	*k*_cat_ (s^−1^)	*k*_cat_/*K*_M_ (L g ^−1^ s^−1^ )
Xyn11-29	877 ± 37	3.30 ± 0.43	708 ± 52	216 ± 17
XynMF13A	1322 ± 5	3.16 ± 0.43	1075 ± 0.75	340

## Data Availability

The sequence data generated in this study were deposited in the EMBL-ENA public database (PRJEB43346 for the first sampling campaign (March) dataset and PRJEB43343 for the second sampling campaign (November) dataset).

## Supplementary Materials

The following are available online at https://www.mdpi.com/article/10.3390/microorganisms9071484/s1: Table S1, List of tagged primers used to amplify expressed genes encoding fungal endo-β-1,4-xylanases (GH11); Table S2, Normalized dataset where the 247,464 fungal GH11 sequences are distributed among the 15 different Operational Functional Units (OFUs) within the sediment samples; Table S3, Mean number of fungal GH11 OFUs (Operational Function Units) detected and diversity indices calculated for oxic and anoxic sediment fractions collected during the wet (March) and dry (November) seasons within two mangrove pristine areas (Avicennia marina and Rhizophora stylosa); Figure S1, SDS-PAGE of the purified Xyn11-29 produced in *P. pastoris*.
